# A new method to experimentally quantify dynamics of initial protein–protein interactions

**DOI:** 10.1038/s42003-024-05914-2

**Published:** 2024-03-12

**Authors:** Babu Reddy Janakaloti Narayanareddy, Nathan Reddy Allipeta, Jun Allard, Steven P. Gross

**Affiliations:** 1 Developmental and Cell Biology, University of California Irvine, Irvine, CA USA; 2 Arcadia High School, Arcadia, CA USA; 3 Department of Mathematics, University of California Irvine, Irvine, CA USA

**Keywords:** Single-molecule biophysics, Optical tweezers, Motor protein function

## Abstract

Cells run on initiation of protein-protein interactions, which are dynamically tuned spatially and temporally to modulate cellular events. This tuning can be physical, such as attaching the protein to a cargo or protein complex, thereby altering its diffusive properties, or modulating the distance between protein pairs, or chemical, by altering the proteins’ conformations (e.g., nucleotide binding state of an enzyme, post-translational modification of a protein, etc.). Because a dynamic and changing subset of proteins in the cell could be in any specific state, ensemble measurements are not ideal—to untangle which of the factors are important, and how, we need single-molecule measurements. Experimentally, until now we have not had good tools to precisely measure initiation of such protein-protein interactions at the single-molecule level. Here, we develop a new method to measure dynamics of initial protein-protein interactions, allowing measurement of how properties such as the distance between proteins, and their tethered length can modulate the rate of interactions. In addition to precise measurement distance dependent motor-MT rebinding dynamics, we demonstrate the use of a dithered optical trap to measure dynamic motor-MT interactions and further discuss the possibilities of this technique being applicable to other systems.

## Abstract (web summary)

This study presents an image processing-based stage height control and optical trap dithering technique to detect protein-protein interactions. It was used to show that the MT binding rate for KIF5B has a strong spatial and motor length dependence.

## Introduction

One of the upcoming challenges in achieving a more quantitative understanding of cellular processes is to develop a single-molecule-level understanding of how protein-protein interactions occur, and how specific factors can tune the dynamics of such interactions (e.g., immune response, and enzymatic activity). In a cell, many of a specific type of protein are present, in a variety of folded conformations, with different post-translational modifications, possibly attached to different sized protein aggregates that alter their diffusion. Further, some may be close to their binding partners, and some may be further away. In this overall cellular ensemble, then, the *Average* behavior of a protein-protein interaction may be very different from what is occurring at the single-molecule level—for instance, there may be some factor that so strongly modifies the probability of an interaction, that almost *ALL* the interactions observed reflect interactions of this subset of proteins affected by the relevant factor. As discussed later, the distance between receptors and their ligands likely tunes cell-cell interactions. To be able to better understand how these interactions initially occur, and how to model them, we thus need to have good single-molecule measurements able to quantify and untangle the effects of different factors.

Understanding protein-protein initial interactions, and how their dynamics are modulated, is central for much of biology, and the technique we present below should be applicable for probing many such systems. After the invention of the single beam optical trap^1^, multiple single molecule studies used optical traps to determine the dynamics of protein-protein interactions once the interactions had formed^2–5^. In most of these studies, the optical traps were built with a focus on determining the parameters related to the energy landscapes of the interaction—e.g., how long the interaction would last as a function of load—but there was less thought about the dynamics of their initiation. Here, however, we apply the optical trap with precise height control to investigate molecular motors, and their interactions with microtubules, to better understand the ‘on rate’, that is, the time it takes for a motor protein to bind to a microtubule, a polymer of tubulin proteins. As an example of some of the features that matter for modulating the protein-protein interactions, we determine how distance between the proteins, and the length of one of the proteins affect the rebinding rate. Further factors, such as the relevance of specific post-translational modifications, would be easily incorporated into the assay. To be able to make such measurements, we need very good control of the distance between the two interaction partners. Below we discuss how this distance is determined and controlled.

Vesicular trafficking inside the cells is carried out on microtubule tracks (MTs) by molecular motors such as kinesins and dynein. This movement is critical for signaling and cell maintenance functions during development. The rates at which cargo carrying motors bind and detach from microtubule determine the overall vesicular distribution and efficiency of cargo reaching their target sites inside the cell cytoplasm. Experimentally, single molecule properties of molecular motors—once they have attached to their filament—have been extensively characterized using optical traps and gliding assays for several decades^6–11^, however we are unaware of any studies using optical traps to directly measure on-rates. The closest we can come are a number of theoretical studies combining theory and experiment, where the rebinding rate of motors functioning as part of a group is inferred via fitting (see e.g^12,13^.). Since the initial binding of a single motor may be different from a rebinding event of motors functioning as part of a group, and in any event in neither case were there clean direct measurements, we set out to make them.

**Fig. 1:**
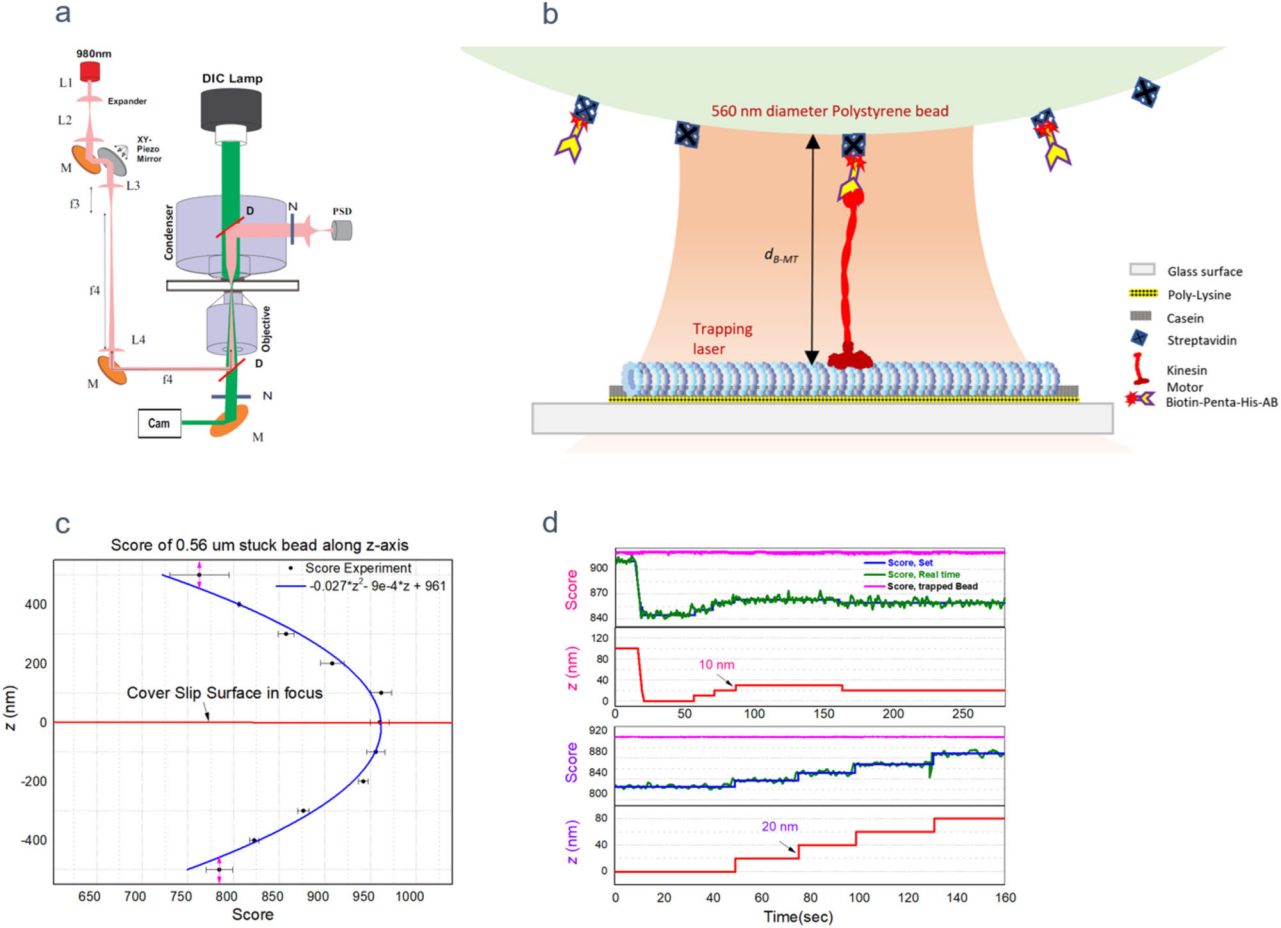
Schematic of the setup and calibration. **a**, **b** Schematic of optical trapping setup and single molecule motor attached bead in the optical trapping system. **c** Experimentally observed bead template match score vs. *d*_*B-MT*_. **d** Figure illustrating the stage movement in steps of 20 nm and 10 nm. Notice the average score for the trapped bead(pink) is nearly constant while that of stuck bead increases every time there is a 20 nm or 10 nm increment (blue line) in z-position of the stage. ‘Score, set’ is the z-position that the stage is set to maintain. It is the average score of the stuck bead 2 s after the z-stage motion.

In our typical in vitro assay, we seek to determine the rate of motor binding to MTs using bead assays in the optical trap (Fig. 1a & b). To do this, purified motors (e.g., kinesin or dynein) are attached to antibody conjugated polystyrene dielectric microspheres (beads) via a specific genetically encoded handle or tag on the end of the motor protein (Fig. 1b). The motor coated beads are held in the optical trap using the microscope designed for simultaneous imaging and manipulation of the bead position. This option helps in studying the protein-protein (motor-tubulin) interactions as the trapped bead with motors on them moves synchronously with the plane of focus.

To determine the on-rate of the motors, the bead (typically with a single motor attached to it) is positioned in the vicinity of a MT, allowing the motor to bind and translocate. At this point, the motor’s MT binding probability is dependent on how far or close the motor is from the MT surface. It’s particularly critical that a known constant distance is maintained during the binding rate measurements, as the cargo-MT spacing directly affects the available tubulin binding sites for the motor binding. The motor binds with certain probability when the average spacing between the MT and the bead surface is in the range of 0–100 nm. Several factors affect the upper limit (for instance, if the extended motor cannot reach the MT, there will be no binding), and it is estimated from the length of the motor, size of the antibody anchor, and magnitude of vertical bead fluctuations in the optical trap at room temperature (RT) after assuming no stage drift. However, stage drift while using a high-resolution video microscope is significant due to RT changes, vibrations induced by air flow from room vents, weight of the sample holder stage etc. This drawback affects the determination of motor rebinding rate because the variation of motor-MT separation due to drifting stage alters the access to MT binding sites. Below, we describe a method we adopted to build a robust optical trapping microscope system with vastly improved stability of MT-bead spacing during the binding rate measurements.

## Results

### Air suspension optical table and Microscope enclosure

As followed in most other studies using optical trapping microscopes, all the components of optical trapping microscope setup, including the laser and optics, were mounted on an air suspension optical table to reduce the mechanical vibrations. We also took an additional step to reduce the air currents and improve the temperature stability by covering the entire setup including the laser beam path with a 6 mm thick dark plexiglass enclosure. Further, the lamp and the controllers were turned on at least one hour before starting the experiment for temperature equilibration. These steps helped minimize the air currents at the sample and improved stability of sample stage and trapping laser.

### Focus locking with autocorrelation of defocused bead template

Several techniques have been proposed for stage drift correction in high-resolution microscopy^14,15^. One of them is a template matching method^16,17^ that employs autocorrelation of a reference image with real time images to estimate the level of matching, specified by the parameter ‘Score’ in labVIEW. The score specifies the accuracy of the match obtained by comparing the template image to the match region using a correlation metric that compares the two regions as a function of their pixel values. A score of 1000 indicates a perfect match, and a score of 0 indicates no match. While using a perfectly focused image as the reference template, the value for the score plateaus when the real time image of bead matches with the template image. It decreases when the real time image drifts away from that reference point as shown in Fig. 1c. Technically the matching is carried out using the intensity cross correlation of the template and real time images. Mathematical form of the cross-correlation of intensities can be found in our previous work^18^. Exploiting this feature, we built a custom LabVIEW program for stage drift correction and to fix various MT-Bead separation distances (also see Methods). The program was custom designed to analyze the video images, acquire PSD signal (force, at 3 kHz) and automatic drift correction (1 s interval) using a XYZ piezo stage.

**Fig. 2:**
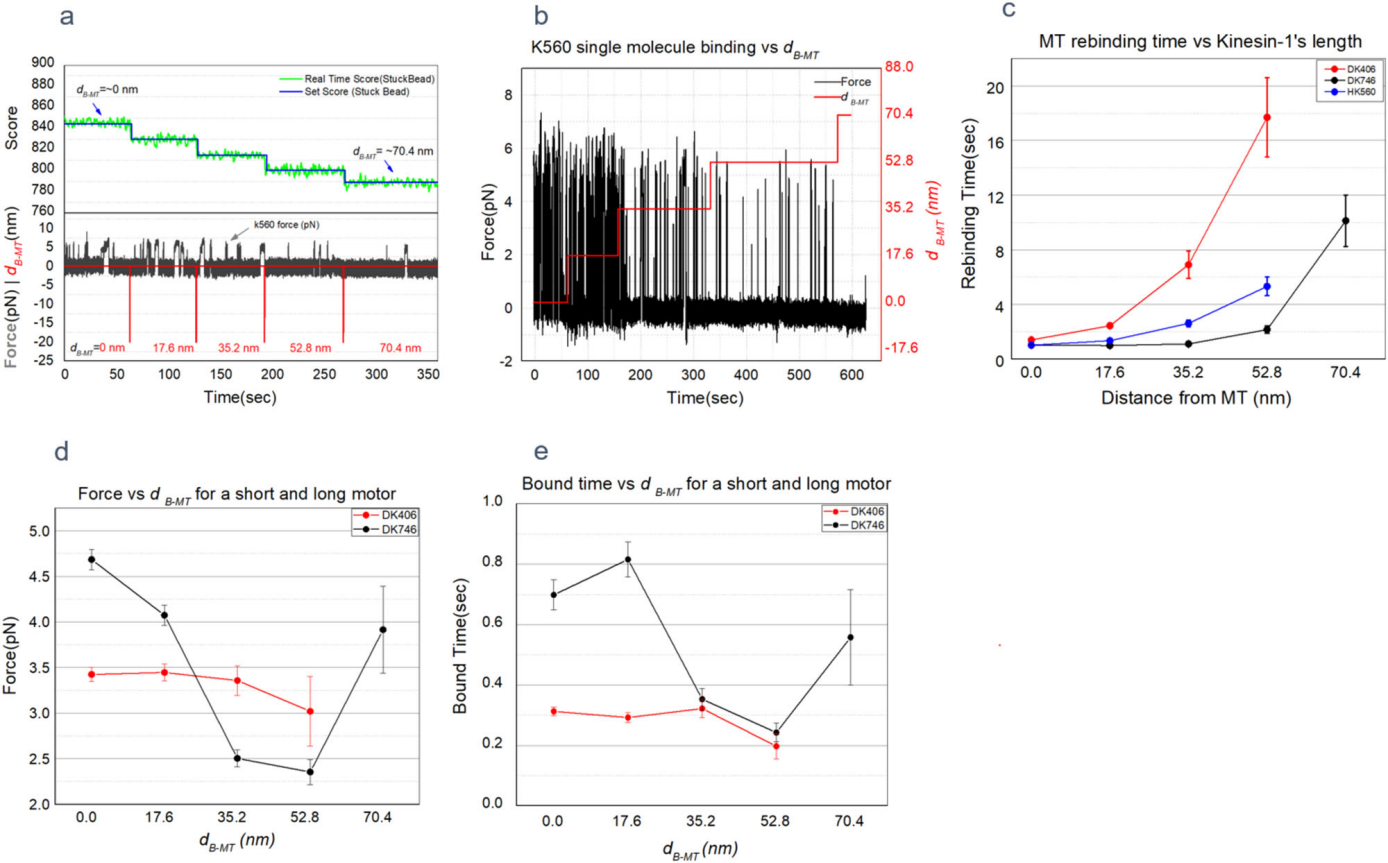
KIF5B (K560) motility parameters with precise stage height control. **a,** **b**. Traces of kinesin binding events as a function of d_B-MT_ recorded after incrementing z-stage in steps of 17.6 nm from the surface with automated drift correction using piezo XYZ-stage (Green and Blue in **a**). **c**. Kinesin rebinding times as a function of distance from the MT for different lengths of kinesin tail (DK406 =Drosophila KIF5B 1–406 amino acids, H-human). **d**. Average peak forces are higher for longer motors, and it decreases as the motors move away from the MT. **e**. Bound time decreases as the motor – MT distance increases. (Averages for 12 single molecule beads, Raw data for 2c-2e can be found in Supplementary Data 1).

For the focus lock the program used pattern learning and matching algorithms to auto-correlate the real time images of a bead (560 nm polystyrene) immobilized on the coverslip with a previously recorded out of focus reference image (of a stuck bead ~300 nm out of focal plane) to estimate the score. The images of stuck bead were registered by DAGE-MTI analog CCD camera and passed through Hamamatsu image processor before acquired by NI-IMAQ frame grabber for digitization. In general, the template match score of stuck bead vs the separation distance *d*_*B-MT*_ exhibits a quadratic behavior (Fig. 1c). As can be inferred from this plot, using out of focus template image has a significant advantage. It vastly improves the real time score change of the stuck bead image at surface vs *d*_*B-MT*_ by pushing the absolute value of the score into linear range where the score change—per nm focus drift—is highest. i.e., if the template or reference image chosen is also that of a bead in perfect focus, the score change—per nm focus drift—gets smaller and smaller near the surface where majority of the binding activity experiments are carried out (Fig. 1c). With the in-focus template bead image, we observed a score change of <0.1% for each 10 nm stage movement as *d*_*B-MT*_ approached to zero (focal plane at the surface of coverslip). In contrast, with the 300 nm out of focus bead template that we used, the score change per nm was as high as 1% for every 10 nm increment near the surface (Figs. 1d and 2a).

### Stable lamp illumination

Stable illumination of the ‘reference bead’ (560 nm polystyrene fiduciary bead immobilized on the coverslip surface to generate match score and position in real time) is crucial because an unstable light source would result in unwanted brightness and contrast variations of real time bead image when template matched with reference image. The score and position of the stuck bead from this pattern matching are used as feedback signal for automatic drift correction of the sample stage and to set the bead-MT separation distance(score). The intensity fluctuations are observed to be higher when the lamps were used for longer than manufacturer recommended lifetime and hence the HBO mercury lamp used for DIC illumination was replaced every 200 h or sooner if there was a noticeable intensity variation in the form of flickering.

### Choosing fiduciary bead size for reference template

Since the intensity pattern of the image varies with bead size, the sensitivity (score change per unit z-motion) could also depend on the size of the reference template bead. To determine which bead provided maximum score change for each 10 nm focus change, we tested three different sizes of reference beads (440 nm, 560 nm, and 800 nm polystyrene beads). Out of the three, 560 nm polystyrene bead turned out to provide highest sensitivity. With a 560 nm polystyrene bead as a template, it was possible to repetitively set and maintain ±10 nm increments of the z-position of the stage at the coverslip (up to ~300 nm above surface). This level of stability in the stage and bead separation combined with optical trap and position sensitive detection system helped us determine the motor-protein and tubulin interactions (see the optical trap dithering section).

### Identifying the surface

One of the major challenges in quantifying the distance dependent protein-protein interactions (in our case, the motor-MT binding rate as a function of separation between them) using an optical trap is that it’s hard to maintain a stable separation (*d*_*B-MT*_, Fig. 1b) between the protein linked bead in the trap and the interacting protein. The difficulty lies in finding a parameter that indicates whenever bead touches the surface (*d*_*B-MT*_ ~ *0*). Technically this parameter should be a measurable feedback signal that varies synchronously with the focus (trap) position. In our case, this problem was solved by tracking the trapped bead (initially in solution, away from the surface) as it is lowered in 10 nm increments and comparing it to a prerecorded out-of-focus image of a similar bead.

We start the process of identifying the surface or *d*_*B-MT*_ = 0 by positioning the center of trapped single motor bead above the MT in such a way that its surface towards the MT is roughly 100 nm deep into the solution without touching the surface. Then the z-piezo stage is used to move the surface up gradually in 10 nm steps towards the trapped bead while simultaneously processing the video to estimate its match score with the reference template. Note that although the coverslip surface is moving relative to the objective, the bead inside the trap stays in focus, so the average score of the trapped bead is nearly constant (Fig. 1d, pink line) till the surface hits the trapped bead. Once the surface touches the trapped bead, moving it further up shifts the trap center below the surface and the bead above or out of focal plane of the objective. This results in altered image contrast in the trapped bead detected as net score change indicating that the surface is within 10 nm from the equilibrium position of the bead. This completes the identification of the surface. Here we start using the match score of the fiduciary bead stuck on the surface within the field of view for drift correction and to maintain the stage at different values of *d*_*B-MT*_ from 0 to 100 nm. We also estimate that due to differences in the refractive indices of the oil and aqueous buffer there is ~12% shift in equilibrium trap/bead position towards the surface. This apparent shift in the set values of *d*_*B-MT*,_ estimated using Snell’s law is proportional to the difference in refractive indices of oil/glass (1.52) and the buffer (1.33). This is in agreement with similar reductions in trapping focus shift (18% towards the surface) for the corresponding stage motion in axial direction has been measured experimentally by K Neuman et al. ^19^.

Note that stuck beads (i.e., reference beads) are crucial for drift correction of the stage and the trapped bead can only be used to identify the surface but not to correct the stage drift when the trap position is inside the solution. This is because the trapped bead is always in the focal plane of the objective thus making its match score insensitive to the stage movement when the trap is inside the solution.

### Motor rebinding rate vs., *d*_*B-MT*_

Biologically, we hypothesize that control of MT-cargo separation could be a mechanism to regulate vesicular traffic, wherein cargos are pushed away or pulled towards the MT, e.g., via conformational changes to the adapter molecules linking the cargo to the MT. The mechanism for this spatial tuning could also be via post translational modifications (PTMs) to the microtubule associated proteins (MAPs), tubulin c-terminal tails, and motors. It’s currently unclear how the cargo MT separation affects the rebinding rate of the motors to the MT. These measurements are challenging, as the distances involved are of the order of tens of nm (combined motor-antibody linkage length ~60 nm). Our system allows the determination of binding rates of kinesin as a function of cargo-MT separation in steps of ~10 nm (Fig. 1d). The experiment was carried out by using truncated human kinesin (k560) purified in vitro after selecting 5 values for ***d***_***B-MT***_ from 0 nm to 70.4 nm each separated by 17.6 nm. We selected 17.6 nm steps (20 nm stage increments corrected due to 12% focus shift) for *d*_*B-MT*_ instead of 10 nm to reduce the experimental time and any possible photodamage caused by the prolonged exposure to trapping laser. As can be noticed from Fig. 2a, b and Suppl. Fig. 1, the duration between the binding events increases with an increase in *d*_*B-MT*_. The results suggest that as *d*_*B-MT*_ is increased from 0 nm to 80 nm (0nm- 70.4 nm after 12% correction), there are fewer and fewer binding events per unit time, suggesting a strong spatial dependence of kinesin-MT rebinding rate.

### Rebinding rate vs tether length

Vesicular cargos are tethered to MTs via motors and MAPs of varying sizes. It’s unclear how the length of the tethers affects the binding probability of the motors to MT. While it is difficult to measure it inside the cells in a clean way due to the noise from other cytosolic factors, it is possible to estimate it in vitro with the precise height control in our setup. Full length of Drosophila KIF5B heavy chain is 980 amino acids(aa) long and broadly consists of motor and tail domains or coiled-coil domains with hinges^20^. KIF5B is mostly conserved across humans and flies with motor domain ~370 aa long and a coiled-coil domain or stalk that extends up to aa 980. Thus, increasing aa count beyond motor domain results in a proportional increase in the stalk length. We purified three different lengths 406, 560, and 746 aa of KIF5B for this on-rate experiment (Drosophila Kinesin 1–406aa, Human Kinesin 1–560aa and Drosophila Kinesin 1-746 aa). All three kinesin constructs have a 6xHIS tag at the end of their c-term which is used to link it to the penta-HIS-biotin antibody-coated streptavidin beads. The on-rate data was collected for 5 different heights from each single motor bead. Measurements were repeated on at least 12 different single molecule beads for each motor construct at ~10–20% bead binding fraction (to ensure a single molecule/bead). In the rare case that a bead had multiple motors, indicated by an obvious second peak in the force traces, these data were excluded. Results shown in Fig. 2c suggest that the shortest motor (DK406) exhibits lowest rebinding rate at the same height in comparison to longer motors.

### Dithering the trap to improve detection of binding events

**Fig. 3:**
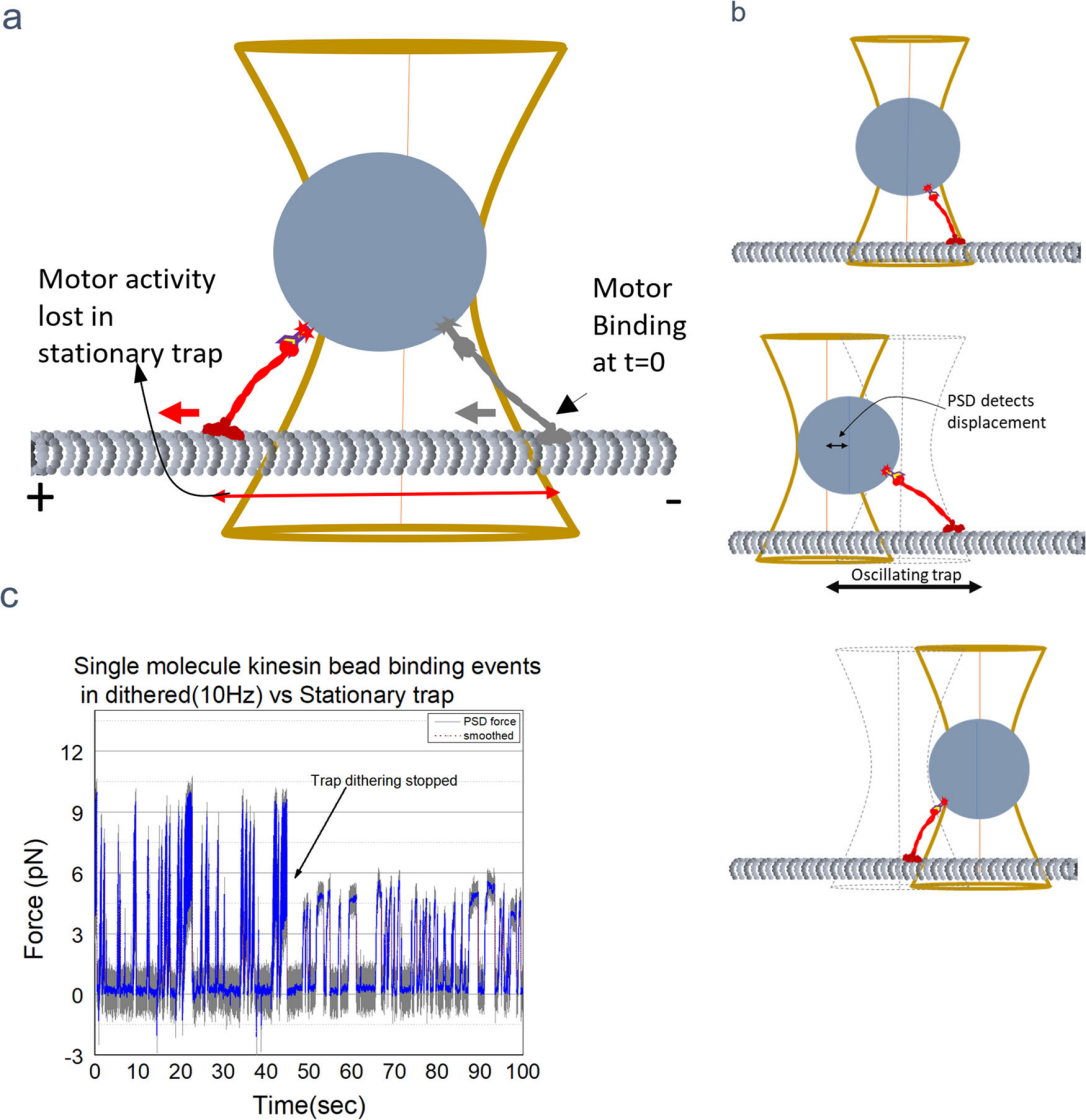
Trap dithering increases the detection of binding events. **a** Illustration demonstrating how motor activity could go undetected until it walks a certain distance to displace the bead from trap center thus resulting in a PSD signal. **b** Schematic showing how moving the trap periodically, ~100 nm both ways result in early detection of binding events. **c** Panel showing traces of kinesin motor binding events with and without trap dithering.

If a binding event is detected via the displacement of the motor linked bead out of the optical trap, some small motor binding events will be missed due to limitations in PSD detection. This is because the motor must travel a certain distance before the bead gets displaced from the center of the trap enough that there is a PSD signal above the baseline. This distance is as high as 120 nm for unidirectional motors like kinesin-560 that has a combined motor antibody linkage length of ~60 nm (Fig. 3a). This could potentially lead to lack of detection of many binding events that do not last longer than the threshold, thus leading to underestimation of rebinding rate. This issue can be minimized by dithering the trap instead of keeping it stationary (Fig. 3b). A trace of motor binding events with and without the periodic displacement of the trap is shown in Fig. 3c. Moving the trap back and forth along the MT axis at a fixed frequency (*f*) leads to detection of all events longer than 1/*f* sec. Note that the dithered trap method has potential to be highly useful in studies of interactions between proteins other than motors. Binding events to substrates such as membranes by non-motor proteins e.g., integrin, could be easily detected with better time resolution than the widely used suction pipette- stationary trap combination^2,5^, due to the trap dithering even though there is no directed motion of the protein coated bead after binding.

**Fig. 4:**
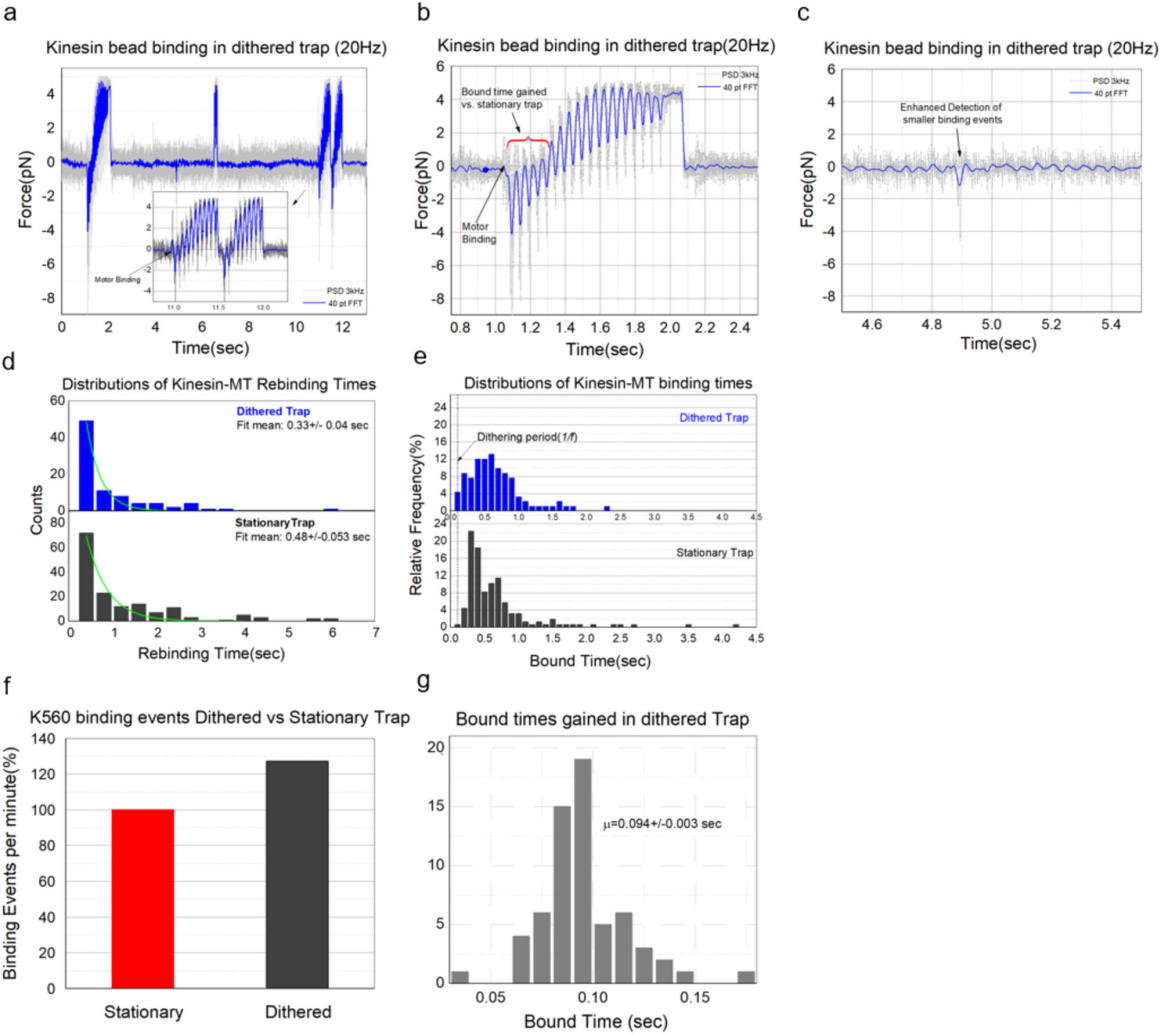
Example traces of kinesin motor in a moving trap. **a** Binding event with trap movement showing gain in the bound time. For this event to be detected in the stationary trap it would have taken 0.2 s, a timepoint when the signal rises above baseline. **b** Another example of binding event showing gain in bound time with dithered trap. **c** Detection of small events of the order of 1/*f* sec is possible with dithered trap. **d** Distributions suggest that the average rebinding time is lower for kinesin motor with dithered trap compared to Stationary trap. **e**, **f** Increased detection of binding events with dithered trap. See the higher relative frequency of detected binding events represented in the first bin ( ~ 1*/f)* for ‘Dithered Trap’ **e**. **g** Histogram of bound time gained with dithered trap extracted from binding events.

Using the trap dithering method, we determined the binding characteristics of the K560 coated single motor beads by moving the trap ~200 nm back and forth along the length of the MT at constant speed (triangular wave, peak to peak 200 nm). During the sweep, the single motor bead was positioned directly above the MT with the *d*_*B-MT*_ set at 20 ± 10 nm and the frequency was fixed at either 10 or 20 Hz. The PSD data acquired at 3 kHz indicates that most binding events could be detected ~0.1 s sooner than possible with a stationary optical trap (Fig. 4a, b, e). Many small binding events lasting smaller than 0.1 s were also detected (Fig. 4a, c). Oscillating the trap during the measurements resulted in an overall increase in the number of detected events, binding duration, and the rebinding rate (Fig. 4d–g). Analyzed results suggest that the number of detected binding events went up by ~27% (Fig. 4f) in comparison to stationary trap method of binding detection. Importantly, this technique is generally useful and could be used to determine transient events (>=1/*f* sec) and stable interactions of both motor and non-motor proteins via the photodiode or any other sensor in the back focal plane of the condenser.

## Discussion

Here we have presented a new method to measure protein-protein dynamics at the single-molecule level. The key features of the method are (1) the careful detection of the surface, (2) the control of the distance of the bead (and its attached protein) relative to that surface, and (3) the detection of the protein-protein binding event due to either the motion of the molecular motor, or the use of dithering to detect when a bond has formed. In the case of motors, the dithering allows more rapid and sensitive detection of binding events, so that ~27% more events are detected. By dithering at 20 Hz, we were able to detect binding events to within ~0.05 s. In principle, one could go even faster. In the case of non-motor proteins, dithering would be essential to detect the binding event.

How do our results compare with what is already known? There are three studies that are most directly comparable to our work. In the first, the rebinding rate of a motor functioning as one of a group to drive microtubule gliding was estimated to be 4.7 /s^12^. In the other two, two motors were attached close together, either to a bead^21^ or using a DNA origami scaffold^22^. In the latter works, the on-rate of the second motor rebinding, while the first motor was still attached, was estimated to be either 0.71/s^21^ or 1.03 /s^22^. So, in the first the average re-binding time was 1/4.7, or 0.21 s, in the second it was 1/0.71 or 1.4 s, and in the third it was 1/1.03 or 0.97 s. Given the discrepancy between the second and third vs the first, it has been postulated that there is negative interference between small numbers of motors^22^. However, our new data—showing that rebinding is affected by distance—likely explains the discrepancy. In the case of a moving MT, where the MT is gliding over surface-attached motors, the surface-MT distance has been measured to be ~17 nm^23^. For our closest approach, our average time between binding (dithered) is 0.33 s (Fig. 4d), consistent within error to the 0.21 s estimate of Leduc et al. In the case of the bead or the DNA origami case, there was only a single motor determining the distance between the motor’s cargo-binding site and the MT. If that motor was relatively extended, the distance could easily be 30 nm or more, which from our data would lead to an expected rebinding time for K560 of 1–2 s (Fig. 2c). So, we suspect that the different magnitude of rebinding times between these different papers likely reflects the role of different geometries in modulating motor on rates and does not require assuming negative interference between the motors. Further, our new data is broadly consistent with past work in the field but may resolve the long-standing apparent disagreement about the value of kinesin’s re-binding time.

By controlling the distance between the bead and the microtubule on the surface, we were able to examine how the rate of binding changes as a function of distance (Fig. 2, Supplementary Data 1). Where might such information about binding as a function of distance yield useful biological insights? While there are likely other examples, we can directly identify two such areas. First, regarding molecular motors, there are several proteins that can alter the cargo-MT distance: MAPs such as Tau have projection domains that can in principle push cargos away from the MT, increasing their distance, and dynactin can bind to the cargo and the MT, forming a tether. In this latter case, the length of the tether would affect the cargo-MT distance, and thus the rate of binding, as shown by our measurements. Second, there is increasing evidence that distance matters in tuning cell-cell interactions. This appears to be true for modulating T-cell receptor interactions^24,25^. It is also relevant for cancer, where “integrin–ECM interactions are tightly coupled to the distances between receptor–ligand pairs”^26^. Significantly, not only are the distances important, but they can be tuned in different ways. In the case of cancer, they are affected by the glycocalyx^26^. Along those lines, another study highlighted that different glycoproteins affect distances, and that “cell surface mucins, such as MUC1 and MUC16, are so consistently upregulated in epithelial cancers that they are considered reliable biomarkers of the disease”^27^. In a slightly different context, a group changed antibody size to change cell-cell distances, and this changed Fc receptor triggering in macrophages^28^. This general question of how to tune cell-cell distances, and the role that this likely plays in function, was also discussed in^29^, where they also highlight its role in the immune system, specifically “size-dependent segregation of proteins has been shown to modulate T cell activation by antigen-presenting cells^30–33^ broaden mast cell and basophil reactivity^34^, and promote antibody-dependent phagocytosis by macrophages^28^, where an antigen height difference of as little as 5 nm can significantly alter phagocytic efficiency.” In conclusion, both for intracellular protein-protein interactions such as molecular motors binding to their filaments, and for cell-cell interactions, the distance between the interactors is likely extremely important for tuning the rate of interactions.

While our experiments highlight the significant effect of distance on on-rates, understanding this from a theoretical perspective is likely to require further study. In the case of cells, such theories are already being developed; see for example^35^. For the case we studied directly, one could imagine a simple model, where the distance to the microtubule affects the number of accessible binding sites reachable by the motor (see Suppl. Fig. 2). Such a model predicts a gradual decrease in available sites as the motor’s distance to the Microtubule increases, and associated with such a decrease, a gradual increase in the amount of time to bind. In actuality, the rate of experimental binding is far more sensitive to distance than might be suggested by such a model (see table, Suppl. Fig. 2), and more sophisticated models will be required to understand it.

While the method is easily applicable to a motor interacting with a filament, several straightforward modifications of the experimental approach are obvious, depending on the questions to be addressed. For e.g., To explore the role of viscosity, one could do similar studies in the presence of fluids with different viscosity. One could explore the impact of attaching the binding partner to different size beads, to determine how altered rotational diffusion affects the protein-protein interaction. Further, to explore how protein density might alter dynamics of binding, one could coat the surface with a binding partner (at some density) and could then bring a bead with the second partner (or partners) close to the surface (as we did above). One could then explicitly determine how alterations in protein density affected binding kinetics. Of course, all these investigations could be carried out on proteins with post-translational modifications, allowing the direct determination of their role in modulating the dynamics of binding. Finally, of course, because one of the binding partners is in the optical trap attached to a bead, one can always also look at the reverse—how does load, or (e.g.,) some other protein in solution, affect the duration of the bond, and its dissipation? An additional variation of interest involves binding of a target to an extended object. Here our “extended object” was the microtubule. However, one could also place a cell (with receptors) on the surface and use the described method to investigate protein-cell interactions, and (e.g.,) how the interactions changed at different locations on the cell surface. It can also be easily extended to a simulated cell surface: imagine attaching several antibodies to the cover slip, and then using them to tether receptors of interest to the coverslip. Then, the receptor’s ligand interaction partner could be attached to the bead, as we have done with the molecular motor, and one could bring the bead close to the surface and measure the time for the ligand-receptor bond to form. If one were interested in making it more realistic to cell membrane, one could do this with an artificial membrane/bilayer just above the coverslip, and have the receptors bound to that instead of directly to the coverslip.

Dithering parameters of peak-to-peak amplitude and frequency were chosen to keep the speed of bead motion close to physiological speeds of vesicular motility (Max Trap speed = Freq * dither distance = 20 s^−1^ *2*100 nm ~4um/sec). Also, for a tether length in the range of 40–80 nm sweeping 200 nm should produce enough displacement from the trap to detect it in the PSD signal. It is unclear whether higher speeds due to increased frequency of dithering affect the binding rate because time scales of protein- protein interactions reported previously could be very short, in the microseconds regime^36^. Nonetheless, the dithering does affect the detected events in three ways (compare histograms Fig. 4d & e). First, as already mentioned, we detect more of the very short events that were missed in the fixed-trap approach (about 27% more). This is the major effect for the distribution of re-binding times. Second, for the distribution of times of bound events, there are two competing effects. Because we detect all events faster (by about 0.1 s), in the dithered data, we would expect to see an increase in slightly longer events as well as the increase in the short events. This expected increase is not immediately obvious, and we believe it is due to a dither-enhanced motor off-rate, due to fluctuating forces on the motor due to the dithering.

In any case, to detect binding events even more rapidly, higher frequencies can in principle be achieved using optical traps assembled with acousto-optic deflectors (AODs). High-speed dithering helps detect interactions of shorter timescales and might be relevant in probing the presence of any metastable states in the system. Although AFM cantilevers with resonance frequencies of the tip oscillations in the range of 100 kHz have been used to study the strengths of protein-protein bonds at different force loading rates^37^ there is no clear evidence on whether and how the rebinding rates are influenced by the relative speeds of ligand and substrate at fixed distances. As far as we know, AFMs have not been used to study the kinetics of initial binding, or re-binding, the focus of this study.

By providing good single-molecule data on the kinetics of binding and unbinding, this method should help efforts to quantitatively model cellular processes, and in turn, should help clarify which of the many factors that in principle can alter dynamics of the protein-protein interactions are indeed the most important. In other words, the non-invasive nature of the dynamic optical trap technique with variable dithering frequency is potentially useful in determining protein-protein interactions of shorter and longer time scales.

In conclusion, we presented a method to measure the dynamics (on-rate) of protein-protein interactions, at the single-molecule level, as a function of the distance between the two proteins. We apply it to molecular motors and find that the binding rate can vary by a factor of 4 or more, as one changes the distance between the motor and the microtubule. Not surprisingly, this on-rate was also affected by the length of the motor, with longer motors having a larger range of distances that they could function at, with minimal impairment to their on-rate. All kinesin motor constructs exhibited higher forces close to the microtubule than when they are farther away (Fig. 2d). When compared to those with shorter tails, longer motors produced higher forces and longer binding duration (Fig. 2e) which showed inverse dependence on the distance between the motor and microtubule. We believe this method requires careful attention to technical details but is relatively straightforward and its implementation to measure a wide range of protein-protein dynamics may benefit multiple research programs.

## Methods

### Optical trap

The optical trapping setup was assembled on an inverted Nikon TE200 microscope using a 980 nm single mode fiber coupled diode laser (EM4 Inc) and optical components (Fig. 1a). Trapping laser beam was passed through an optical isolator to reduce the back reflections from destabilizing the power. For the experiments with kinesin, the laser power was set to achieve a trap stiffness, k_*Trap*_, of ∼0.045 pN nm^−1^ while using the polystyrene bead of 0.56 μm (streptavidin conjugated, Spherotech).

### Motility assays

Single motor experiments were carried out in the motility buffer (80 mM Pipes pH 6.9, 50 mM CH3COOK, 4 mM MgSO4, 1 mM DTT, 1 mm EGTA, 10 μM Taxol, 1 mg mL^−1^ casein). In all the rebinding rate assays, single motor kinesin coated polystyrene beads were prepared just before the measurements. The motors DK-406-His/K-560-His/DK-746-His (Kinesin-1, aa 1-560/Drosophila Kinesin aa 1–406/ Drosophila Kinesin aa 1–746; His tag at c-term) were diluted to ∼20 nM before mixing with ∼1 pM of biotinylated penta-His- antibody conjugated streptavidin beads stored at 4 C. This ratio produced the bead binding fraction of 10–20% and was maintained to maximize the probability of finding single motor beads in the solution. The bead-motor incubation (∼50 μL volume) was carried out at room temperature for 10 min. At the end of incubation, sample chamber with preassembled microtubules was washed with ∼50 μL of warm filtered buffer just before injecting the incubated mixture. Experiments were carried out at RT in motility buffer supplemented with 2 mM ATP and oxygen-scavenging system (0.25 mg ml^−1^ glucose oxidase, 30 μg ml^−1^ catalase, 4.5 mg ml^−1^ glucose).

In general, small dust or debris in the buffer solution gets pulled into the trap along with the bead. Trapped dust interferes with binding of motor to MTs. To prevent this large dust particles and aggregates of casein in the motility buffer were removed using a 100 nm centrifugal filter (Millipore).

### Cloning and protein purification

DK406 and DK980 plasmids were procured from Addgene (plasmid ID #129764 and #129762, generously deposited by William Hancock lab). DK746 was cloned in house starting from full-length DK980 by following general protocols of restriction enzyme digestion method. To clone DK746, the amino acid sequence from 741 (SacI enzyme cleaving site) to 974 was excluded from the DK980 plasmid by PCR amplification of region of interest using 5′-aaagagctccttgttccgcgtgggagccat-3′-F and 5′-aaagagctcggacacctgccgggtgtggg-3′-R custom designed primers (IDT). A clean PCR product was digested with SacI enzyme, cleaves gag-ctc to generate the sticky ends and cleaned again with gel extraction before proceeding to ligation with T4 DNA ligase. The ligated product was transformed into DH5alpha and placed on antibiotic treated agar plates at 37 C overnight to get single colonies for the DK746 plasmid miniprep (All enzymes, DH5alpha, and the PCR kit used for cloning were procured from New England Biolabs).

Kinesin constructs used in this study were purified via affinity chromatography (using Ni-resin, Fisher Scientific) using a 6×HIS tag at their tail as described earlier^38^. All proteins were expressed in 500 ml of Rosetta bacterial cultures in Terrific Broth. Induction of protein expression was carried out by adding 1 mM IPTG to the culture at ~0.8 OD and the temperature was maintained at 18–20 °C for 24–36 h (agitation at 180 rpm). Pelleted cells were lysed in 35 ml wash buffer (50 mM Na2HPO4, 300 mM NaCl, and 75 mM Imidazole, PH 7.4) using ultra sonication on ice bath. Lysates were clarified by centrifugation at 4 C, 14000 rpm for 60 min to collect supernatant. The filtered supernatant (0.2 µm, syringe filter) was incubated with Ni-Resin for 1 h at 4 °C on a rotor before loading into a column for washing and elution. Elution was carried out in the wash buffer with 300 mM Imidazole and dialyzed to reduce the imidazole concentration before storing at −80 °C for long-term use. A second level of affinity purification was carried out using MT binding with 1 mM AMP-PNP and release with excess 5 mM ATP before using the protein for single molecule experiments.

### Stage drift correction and *d*_*B-MT*_ setting

Template matching in our case required a reference image of the defocused bead for comparison. For that purpose, functionalized polystyrene beads ( ~ 560 nm) were immobilized on the polylysine coated coverslip surface used to construct sample chamber where the MTs were also attached. Post MT attachment and flushing of unbound MTs, the fiduciary beads were flown into the chamber for attachment to polylysine surface. This was followed by blocking of the surface with 5 mg/ml casein in 35 mM pipe buffer supplemented with 4 mM EGTA and 1 mM GTP. The wait time of incubation between the steps is 20 min.

Immediately before the experiment, a 40 × 40 pixels size template image of the attached fiduciary bead is selected from a previously recorded Image. The search for this template pattern is carried out in the specified region of interest (ROI) of real time image from the camera. The ROI selected should have a fiduciary bead to restrict the search area and reduce the computing time during the real time data acquisition. Match_patten.vi (in combination with setup_learn_pattern.vi, learn_pattern.vi, and setup_match_pattern.vi) from LabVIEW software and compatible NI image acquisition boards installed in a computer, provides template match score and position co-ordinates of the stuck bead in real time at ~25 Hz. The above parameters of the bead were fixed for kinesin-MT rebinding rate measurement at each *d*_*B-MT*_.

Inside the program, gray scale values of the reference template image are compared with those from the real time image in the ROI. The vi stores intensity patterns or curves from template image at the start of the program and cross correlates the stored patterns with the real time image in the search area. The program is written in such a way that the ROI is selectable using mouse click on the video. To optimize the speed of processing, ROI is restricted to 2x the size of the template image. The program is configured to search for one match with a minimum degree of matching threshold to exclude the noise. The degree of gray scale intensity match is indicated by a quantity called Score. Its values range from 0 to 1000 for 0% match to 100% match with the template respectively.

Stage drift during the measurement reflected in direct changes to the score and position coordinates. This unwanted drift was corrected, every 1 s, during the measurement by using an automated piezoelectric XYZ stage. To set *d*_*B-MT*_ first, the surface is identified and then stage is moved by 100 nm in the z-direction to measure the score change/nm slope. This slope is used to set and maintain the *d*_*B-MT*_ = 0 and each subsequent 17.6 nm increments from the surface.

### Scoring criteria for binding events

The data collection for single molecule kinesin motility experiments were carried out as described in our previous studies^39^. Force data is collected at 3 kHz using a position-sensitive detector placed in the back focal plane of the oil-based condenser and filtered using a 40 pt FFT function. A binding event is scored when the force signal crosses a threshold force of 1 pN with an additional criterion that the event lasts for >= 10 msec above the threshold force. This limit of 10 msec was arrived at to eliminate the false detection of sporadic noise as binding events. Detachments of motors were identified using the sudden slope changes observed in the force signal. ‘Rebinding time’, which is used to calculate the number of rebinding events per unit time or rate is the duration between a detachment and rebinding events detected using a threshold. In other words, it is the time between detachment (marked by a sudden fall in the force signal towards baseline) and the next binding event. Similarly, bound time is the duration of activity where the force signal stays above the threshold force immediately before a detachment occurs.

### Trap dithering implementation

Trap oscillation was carried out by placing a piezo-electric mirror in the path of the laser trapping beam. The piezoelectric mirror mount and controller which can oscillate the mirror at up to 50 Hz was procured from Mad City labs. This mirror was placed in between the lenses L2 and L3 at their common focal point (Fig. 1a). L3 is the lens frequently used for manual trap steering in the focal plane. Periodic dithering of the trap in the form of triangular wave at the desired frequency *f* was achieved by applying an analog voltage signal from NI-PCIe 6731 DAC channel to the piezo mirror controller. The amplitude of the voltage was adjusted to achieve a ±100 nm displacement of the trapped bead from the initial position. Real-time DIC images were acquired by NI frame grabber board PCI-1410 and preprocessed using Hamamatsu image processor to subtract the background and enhance contrast. During a single molecule experiment with dithering, the single motor bead inside the trap was positioned directly above the MT, at *d*_*B-MT*_ ~ 20 nm. As in stationary trap kinesin binding experiments, a real-time stage drift correction was applied to the stage in X, Y, and Z directions during the measurements.

### Reporting summary

Further information on research design is available in the Nature Portfolio Reporting Summary linked to this article.

### Supplementary information

Supplementary Figs.

Description of Additional Supplementary Files

Supplementary Data 1

Reporting Summary

## Data Availability

The raw data for the graphs in Fig. 2c–e can be found in Supplementary Data 1. All other data are available from the corresponding author on reasonable request.
