# Supplementary material for: A new method to experimentally quantify dynamics of initial protein–protein interactions
Source: Commun Biol. 2024 Mar 12;7:311. doi: 10.1038/s42003-024-05914-2 (PMC10933273; doi:10.1038/s42003-024-05914-2)
Supplement: Supplementary file 1 — Supplementary Figs. [file 42003_2024_5914_MOESM1_ESM.pdf]

Supplemental Information: Additional example trace of height vs MT rebinding rate

Supplementary Fig.1. K560 motility parameters with precise stage height control. Trace of kinesin binding events as a function of $d_{B\text{-}MT}$ recorded after incrementing z-stage in steps of 20 nm from the surface with automated drift correction using piezo XYZ-stage. Note that binding frequency decreases initially with increase in distance and gets restored as the distance reduces to zero, suggesting that the change in binding rate is due to distance rather than optical damage. Quantitation (not shown) of binding rates at the same z-distance taken at different times shows similar binding rate (to within experimental error) with no obvious trend of slower binding at later times in the experiment.

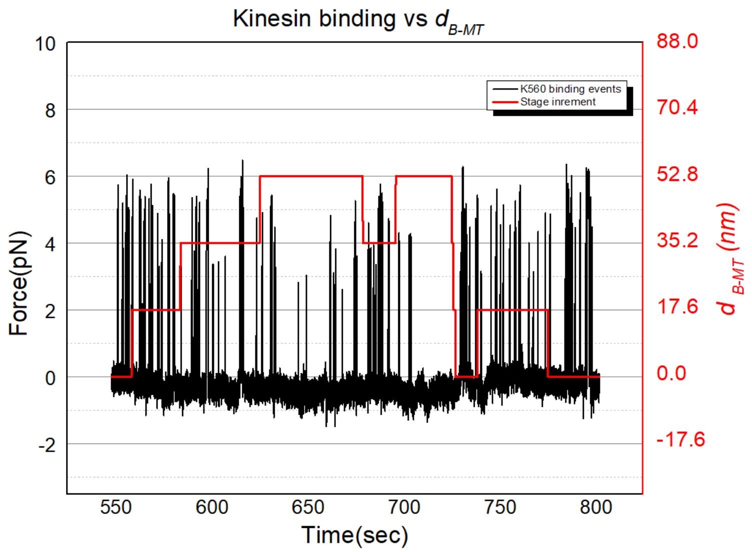

Supplemental Information: Attempted model to explain the dependence of $d_{B\text{-}MT}$ on MT rebinding rate

Supplementary Fig.2. A simple model of reduction in accessible binding sites as the motor moves away from the MT doesn't account for the experimentally observed rebinding time changes presented in the table.
Assumptions: L- length of the motor, s – MT surface accessible for motor up to this distance. δs – Inaccessible fraction of MT surface for the motor to bind as the bead-microtubule distance increases. Predicted rebinding time from model = $1/(1-\delta s)$.

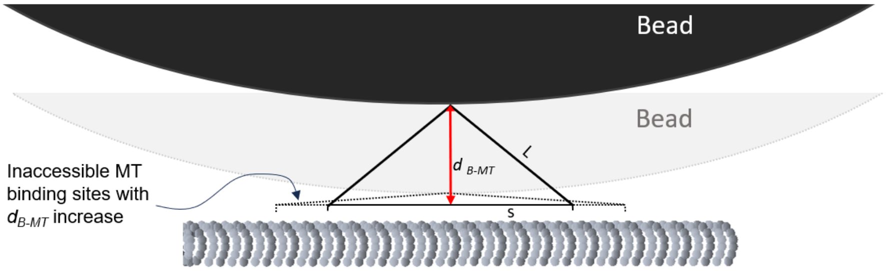

$d_{B\text{-}MT}$ (nm)	$S=\mathrm{sqrt}(L^2-d_{B\text{-}MT}^2)$ (nm) for L= 80 nm, 70 nm and 60 nm	Reduction in binding sites vs. $d_{B\text{-}MT}$ (δs)	Predicted Rebinding time increase, Model	Rebinding time increase from $d_{B\text{-}MT}$=0 nm, Experiment
17.6	78<br>68<br>57	0.025x<br>0.03x<br>0.05x	1.025x<br>1.03x<br>1.05x	0.90x<br>1.34x<br>1.69x
35.2	72<br>60.5<br>48.5	0.1x<br>0.14x<br>0.19x	1.11x<br>1.16x<br>1.23x	1.1x<br>2.6x<br>4.9x
52.8	60.1<br>46<br>28.4	0.25x<br>0.34x<br>0.53x	1.33x<br>1.52x<br>1.88x	2.2x<br>5.3x<br>14x
61.6	51.5	0.35x	1.53x	6.0x

Reduced Focus shift is corrected due to mismatch in RIs
