# Supplementary material for: A new method to experimentally quantify dynamics of initial protein–protein interactions
Source: Commun Biol. 2024 Mar 12;7:311. doi: 10.1038/s42003-024-05914-2 (PMC10933273; doi:10.1038/s42003-024-05914-2)
Supplement: Supplementary file 2 — Description of Additional Supplementary Files [file 42003_2024_5914_MOESM2_ESM.pdf]

Description of Additional Supplementary Files

File name: Supplementary Data 1
Description: Raw data for panels in Fig.2c- 2e.
